# Creatine in women’s health: bridging the gap from menstruation through pregnancy to menopause

**DOI:** 10.1080/15502783.2025.2502094

**Published:** 2025-05-15

**Authors:** Abbie E. Smith-Ryan, Gabrielle M. DelBiondo, Ann F. Brown, Susan M. Kleiner, Nhi T. Tran, Stacey J. Ellery

**Affiliations:** a University of North Carolina at Chapel Hill, Applied Physiology Laboratory, Department of Exercise and Sport Science, Chapel Hill, NC, USA; b University of Idaho, Human Performance Laboratory, Exercise, Sport and Health Sciences, Moscow, ID, USA; c High Performance Nutrition, LLC, Mercer Island, WA, USA; d Hudson Institute of Medical Research, The Ritchie Centre, Clayton, Australia; e Monash University, Department of Obstetrics and Gynecology, Clayton, Australia

**Keywords:** Creatine monohydrate, female, physiology, nutrition, sleep, perimenopause

## Abstract

**Background:**

Creatine supplementation in women has gained attention for its potential benefits beyond muscle growth, including reproductive health, cognitive health and aging. Women exhibit distinct physiological differences from men, influenced by hormonal fluctuations during pre-menopause, pregnancy, and menopause, and these factors should be considered for their influence on creatine metabolism.

**Objective:**

This review aims to provide a historical evaluation of creatine supplementation in women, its potential applications across female-specific life stages, recent research highlights, and targets for future research. The review also considers the impact of hormonal changes on creatine metabolism and effectiveness as a dietary supplementation.

**Methods:**

This is a narrative overview of historical and recent research evaluating the effects of creatine in women.

**Results:**

Early studies demonstrated the benefits of creatine on exercise performance in women, though they often overlooked menstrual cycle variability. Recent research has begun to account for these hormonal fluctuations, enhancing the understanding of creatine’s applications. Creatine supplementation has shown positive effects on muscle strength, exercise performance, and body composition, particularly when combined with resistance training. Additionally, creatine may improve mood and cognitive function, potentially alleviating symptoms of depression. Emerging evidence suggests creatine’s benefits during pregnancy and post-menopause, though data on perimenopausal women remains limited.

**Conclusion:**

Creatine supplementation presents a promising strategy for enhancing various aspects of women’s health across the lifespan. Future research should focus on optimizing dosing strategies, understanding long-term health implications, and exploring creatine’s effects during pregnancy and perimenopause.

## 1. Introduction

**Figure 1.**
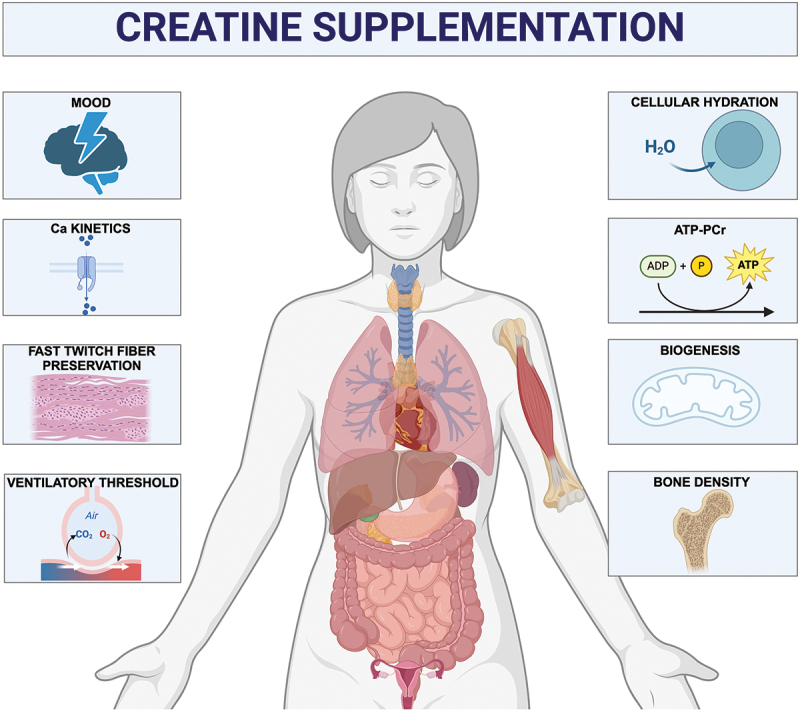
Theoretical model for the implications for creatine supplementation in females specifically.

The use of creatine dietary supplements continues to grow, partly driven by its expanding applications beyond muscle growth to include cognitive health, aging, and women’s health. Recent research has highlighted the potential application and benefits of creatine for women, spanning various life stages and physiological conditions. Women exhibit distinct physiological differences from men, driven primarily by the cyclical fluctuations of sex hormones in pre-menopause and pregnancy, and the unpredictable hormonal changes associated with the menopause transition. Although women are the primary consumers of dietary supplements in the U.S., the use of creatine in women has only recently gained traction due to its pleiotropic effects on health. The growing attention given to creatine use in women is important due to the known physiological sex differences between males and females, as it relates to creatine synthesis [1], with some reports demonstrating 20% lower synthesis rates, as well as a 30–40% lower dietary creatine intake on average [1]. Further sex differences in bioenergetics, as well as differences in fluid hemodynamics, may be key targets for creatine supplementation use in women (Figure 1). Research suggests that creatine metabolism supports bone density, cognitive function, and metabolic wellness, making creatine supplements potentially beneficial for women at various life stages [2]. The current brief review aims to highlight the historical evaluation of creatine supplementation in women, the potential for use in female-specific life stages, and targets for future research in this space. This review also leverages our previous comprehensive reviews on the topic [3,4] and highlights groundbreaking research in women.

### 1.1. Highlighting the foundation: creatine’s unique application in women across the lifespan

**Figure 2.**
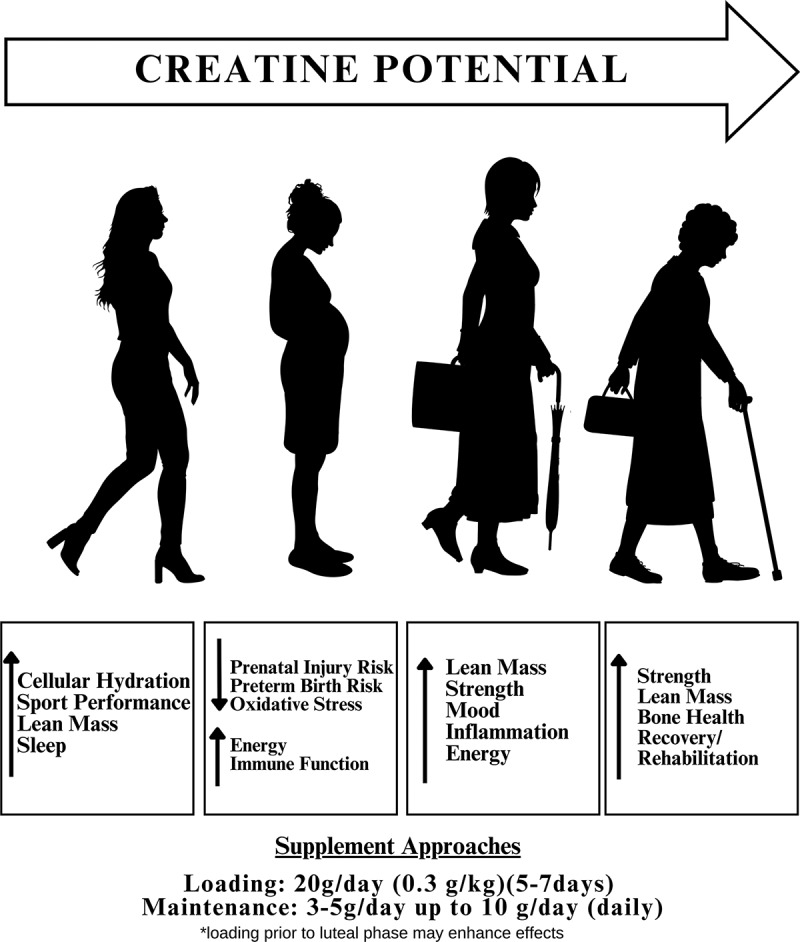
Creatine supplementation may have various targeted effects, dependent upon life stage.

Collectively, research suggests that women may benefit from creatine supplementation in a similar effect reported in men, with positive impacts on exercise strength performance, sport performance, and fatigue. We have previously comprehensively reviewed the mechanistic potential for creatine use in women and the impact of female reproductive hormones [3,4]. We also highlighted the potential benefits of creatine in women in pregnancy and mood (Figure 2).

In 2016, Ellery et al. [3] wrote a comprehensive review describing sex-based differences in creatine synthesis and storage that provided evidence for its importance in women’s health. The paper details how hormonal changes through the menstrual cycle, pregnancy, and menopause can influence creatine synthesis, transport, and creatine kinase expression while also impacting the potential effectiveness of creatine supplements. This paper was the first to propose “creatine as an essential dietary metabolite of pregnancy,” describing the role of creatine metabolism for placental health and fetal growth and metabolism. Ellery and fellow authors also highlighted the potential benefit of creatine supplementation for enhancing high-intensity short-duration exercise, and its use to alleviate symptoms of depression, particularly in women. Ultimately, this review clearly describes that males and females store, metabolize and use creatine differently, while also highlighting the need for larger more longitudinal evaluations of creatine for women.

In 2021, Smith-Ryan and team [4] comprehensively collated existing research exploring the effects of creatine supplementation in women, reporting a positive effect on strength, exercise, and sports performance. Other potential health applications were highlighted related to mood and cognition, as well as positive effects during different female-life stages, such as pregnancy and post-menopausal. The paper also highlighted the advances in research for the potential benefits of supplementation on bone health, when combined with resistance training in post-menopausal women. This paper also summarized potential supplementation doses and considerations to support physical performance, muscle health, and cognitive function, particularly when accounting for variations in creatine metabolism driven by female hormones.

### 1.2. Women have purchasing power

Creatine use and sales continue to rise, increasing 120% (~$20 million) from 2021 to 2022 [5], with a projected exponential growth into 2030 [6]. This increased growth is primarily a result of an expanded consumer base beyond the typical male gym-goers, whereby women are now major contributors to this expanded market. Greater evidence and knowledge of the use of creatine beyond physical performance [5]; have also contributed to this expanded market. It is well known that the largest consumers of dietary supplements are women (CRN), with a considerable motivation for purchase embedded in improving health. Leveraging expanding scientific evidence on the applications of creatine beyond exercise performance and positioning creatine as a preventive health measure rather than solely as a sports-specific supplement has expanded its appeal, particularly when framed as a tool for long-term vitality, strength, and resilience. In combination with increased education and marketing, innovative consumer-friendly formulations – such as gummies and on-the-go stick-packs – enhance convenience and accessibility, increasing sales and use among women. Marketing strategies rooted in behavioral science have supported the rise in consumer perception and adoption of creatine. The “Theory of Planned Behavior” and “Behavioral Economics” suggest that framing creatine supplements as a means to prevent future health costs encourages consumer investment. The “Elaboration Likelihood Model” underscores the need for a dual marketing approach, leveraging scientific credibility and emotional appeal to engage different consumer segments [7–9]. The industry has leveraged scientific evidence of creatine’s impact on women’s health during life stage transitions – including its role in pregnancy, menopause, and sports performance – reinforcing credibility and driving long-term consumer trust. Creatine utilization among women will continue to rise as the research in this space continues to expand. Indeed, this includes research in targeted life stages, such as pregnancy and perimenopause, as well as evaluating the combined application of creatine and other evidence-based nutritional supplements.

### 1.3. A timeline of women-focused creatine research

**Figure 3.**
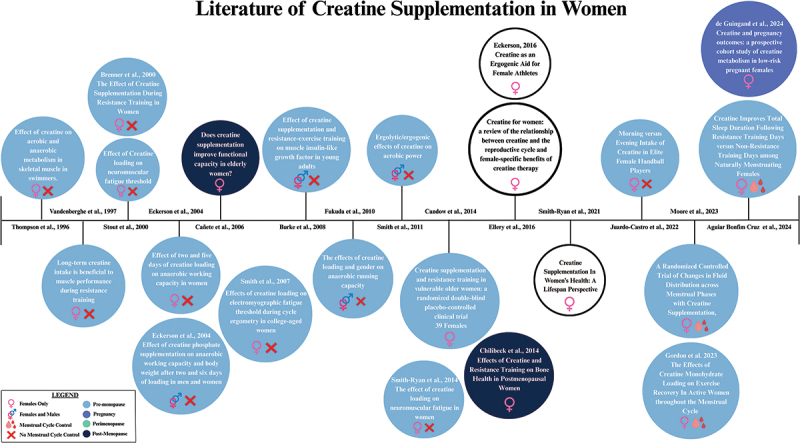
Timeline of key peer-reviewed manuscripts highlighting the benefits of creatine in females for strength, sport performance, and/or health. Narrative review manuscripts are depicted by outlined circles and were not considered for menstrual cycle control. Notably, to our knowledge, there have been no studies examining creatine in perimenopausal women. In Chilibeck’s study focusing on post-menopause, the average age was 57 ± 6 years, which overlaps with the upper range of perimenopause. Further, literature on the effects of creatine in pregnancy exists, but mostly in animal models. This timeline highlights the need for more menstrual cycle-controlled studies examining the effects of creatine in pre-menopause and the gap of literature in pregnancy and perimenopause.

Creatine supplementation in females has been studied for several decades, initially focusing on exercise performance in active females and extending to older women (Figure 3). Early research, such as the studies by Thompson et al. [10], explored 2 g creatine per day in female swimmers. Vandenberghe et al. [11] evaluated 10 g creatine per day combined with resistance training in sedentary females. Additional studies in the early 2000s explored the effects of creatine supplementation on exercise performance, demonstrating benefits, but often overlooked the variability in menstrual cycle hormones. Studies in older women, like those Cañete et al. [12] and Chilibeck et al. [13], have shown improvements in functional capacity and muscle performance. More recent research, including studies by Gordon et al. [14], Moore et al. [15] and Aguiar Bonfim Cruz et al. [16], has started to account for menstrual cycle phases, enhancing the understanding and application of creatine supplementation across different stages of women’s health. Human research in pregnancy is also now being explored (de Guingand et al. [17]), filling a significant evidence gap for this important life stage. To date, data among perimenopausal women remains lacking and is a key priority of future research. The progression of research across the last two decades highlights the evolving focus on optimizing creatine use for various life stages and health outcomes in women.

## 2. Hormonal milestones and creatine

### 2.1. Creatine during the reproductive years

Current research suggests that creatine supplementation has a multitude of benefits, specifically in premenopause. The menstrual cycle consists of two distinct phases: the follicular phase and the luteal phase. These phases are marked by fluctuations primarily in estrogen and progesterone, which drive many of the physiological changes experienced during the cycle. These changes in hormone levels may also impact creatine metabolism, including endogenous creatine synthesis, transport, creatine kinase kinetics, and bioavailability over time [3]. Additionally, rodent models have shown that creatine kinase activity aligns similarly to the fluctuations in estrogen that occur during the menstrual cycle [18]. More recently, population data has demonstrated suboptimal dietary creatine intake in women (<13 mg/kg per day); inadequate dietary creatine intake was associated with a higher risk for oligomenorrhea, pelvic infection, hysterectomy, and oophorectomy [19].

Due to sizable menstrual cycle variability, hormonal contraceptive use, hormone variability through pregnancy and perimenopause, and likely inadequate consumption of dietary creatine, future research exploring the impact of these life stages on creatine metabolism is essential. Creatine supplementation, widely recognized for its performance-enhancing benefits, supports high-intensity exercise by saturating phosphocreatine stores, acting as an ergogenic aid and delaying fatigue [20,21]. Creatine as a performance-enhancing supplement in women has been well studied and demonstrates similar performance benefits as reported in men [4,22]. More recently, due to the potential for alterations in performance across the menstrual cycle and reported changes in the perception of fatigue [14], controlling for the cycle along with creatine supplementation has been an important step forward with creatine application.

### 2.2. Spotlight on recent breakthroughs in creatine research in women

While creatine supplementation in females has been explored over the last two decades, particularly as it relates to performance, only recently has the menstrual cycle been accounted for. Due to metabolic and physiological changes that are altered across a normal menstrual cycle and with exercise [23], accounting for hormonal changes and menstrual cycle phases when exploring the implications for creatine supplementation is essential. A few recent studies have highlighted the effects of creatine in females when accounting for the menstrual cycle and hormonal phase [14–16].

#### 2.2.1. Creatine and cellular hydration in females

Creatine’s molecular structure allows for binding water molecules and shuttling this water from the extracellular fluid to intracellular space. Cyclical changes across the menstrual cycle, particularly in the luteal phase (LP) cause shifts in fluid with more extracellular fluid retention, often leading to women reporting feelings of bloating. Previous studies have shown that creatine supplementation can improve total body water by shifting extracellular fluid into the cell [15]. In a recent randomized controlled trial evaluating the effect of creatine monohydrate loading versus placebo across the menstrual cycle, researchers reported significantly greater volume of fluid in total body water (TBW), extracellular fluid (ECF) and intracellular fluid (ICF) following creatine supplementation [15]. These changes were seen in the LP, regardless of hormonal contraceptive usage and without changes in body weight, indicating the creatine monohydrate loading may be beneficial in promoting cellular hydration, particularly in the LP.

Phase angle (PhA), measured via bioelectrical impedance spectroscopy (BIS), is a measure of cellular health and hydration status [24]. PhA is measured via the reactance and resistance of tissues during BIS [24]. In a recent study evaluating the effect of creatine loading (20 g/day for 5 days) versus a non-caloric placebo on PhA across the menstrual cycle, the group consuming creatine reported significantly greater PhA during the follicular phase (FP) (Pre-Post Cr supplementation: +0.18 ± 0.08°) and in the luteal phase after 5 days of loading, when compared to placebo (Cr-PL: 0.37 ± 0.17°), indicating the benefit of creatine on cellular integrity [25]. The improvements in the follicular phase are possibly impacted by the loss of erythrocytes and iron seen with menstruation and the luteal-phase effects impacted by improving cellular fluid balance [25]. Collectively, it appears creatine may be an especially effective intervention for improving cellular health and integrity during menstruation and in the luteal phase. Future research should explore the impact of these fluid kinetics and the effects on dehydration, performance with environmental stressors, and with aging and clinical conditions that would benefit from improved cellular integrity and phase angle.

#### 2.2.2. Creatine effects on sleep and mood

The application of creatine supplementation for brain health is an important opportunity for women, as they face key challenges associated with sleep hygiene and mental health linked to hormones. New research suggests creatine supplementation in females may support aspects of brain health, including sleep. Sleep disturbances are a significant factor in the mental health challenges faced by young females, with poor sleep often exacerbating anxiety, depression, and body image concerns [26–28]. During puberty and premenopause, body image concerns intensify, often leading to higher rates of anxiety and depression among females compared to their male counterparts [29]. These pressures, compounded by hormonal fluctuations, can disrupt sleep patterns, exacerbating mood disorders and overall well-being. Although disrupted sleep has been associated with altered menstrual cycle phases, other data suggest sleep throughout the menstrual cycle does not differ by phase [30–34]. Regardless of the menstrual cycle phase, resistance training has been clearly identified as a lifestyle behavior that positively influences sleep quality and quantity [35–38]. Interestingly, there is accumulating research showing that creatine supplementation has favorable effects on the indices of brain health and function with the potential to impact sleep [39–41]. Specifically, creatine supplementation may affect cognitive processes related to sleep deprivation, which could have important implications for sleep quality, continuity, and quantity [39,40]. Recent research has demonstrated increased total sleep duration among naturally menstruating females following resistance training days [16]. Although the underlying mechanism remains unknown, creatine supplementation contributes to enhanced brain energy metabolism and therefore may promote longer sleep cycles. By supporting sleep regulation, creatine supplementation may offer a novel approach to improving mental health outcomes in young females. Better sleep not only improves mood but also strengthens emotional resilience, potentially reducing the impact of mental health challenges. This emerging evidence positions creatine as a promising tool in addressing the dual issues of sleep disturbance and mental health concerns in this population.

### 2.3. Creatine in pregnancy and perinatal brain injury

**Figure 4.**
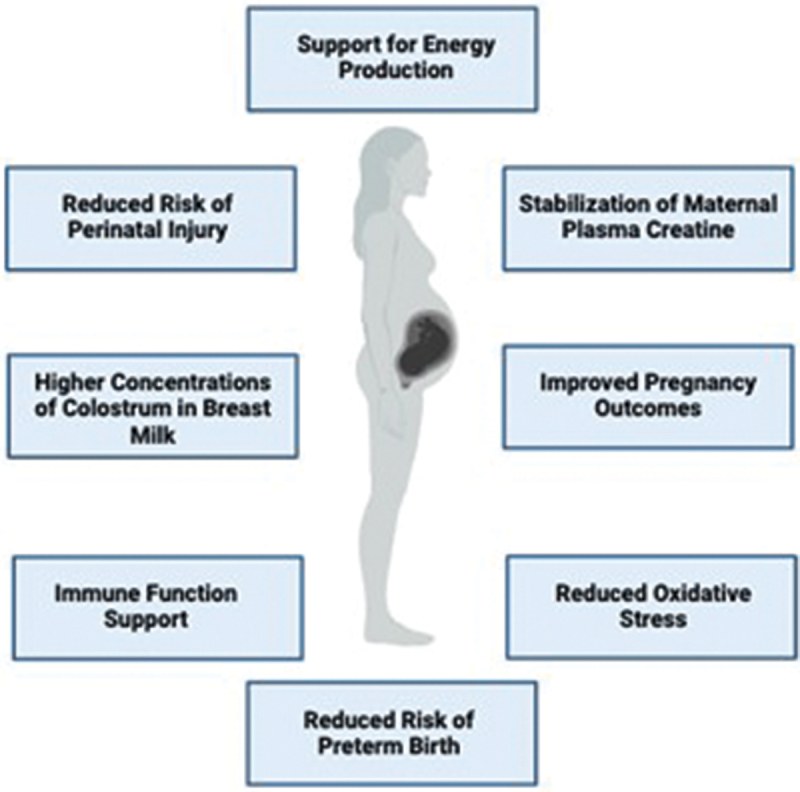
Theoretical model for impact of creatine intake during pregnancy.

Whether creatine should be considered a conditionally essential nutrient for a successful pregnancy is under investigation. Evidence is growing demonstrating alterations in creatine homeostasis throughout healthy human pregnancy, as well as during pregnancy complications associated with hypoxia or restricted nutrient delivery to the developing fetus (Figure 4). Given the emerging data on the importance of creatine throughout pregnancy and apparent associations between dietary animal protein intake and maternal plasma creatine concentrations [17], questions have arisen about whether pregnant populations consume adequate amounts of dietary creatine and what effects suboptimal amounts may have on reproductive and pregnancy outcomes. Sub-population analyses from the 2017–2020 National Health and Nutrition Examination Survey (NHANES) found approximately 6 out of 10 pregnant women in the US (57.2%) consumed creatine below the recommended amounts for an adult female, suggesting a possible risk of creatine malnutrition in this population [42]. Indeed, an evaluation of the NHANES data found that women who consumed ≥13 mg of creatine per kg body mass daily were at a lower risk of obstetric conditions compared to those who consumed <13 mg of creatine daily [19]. Adequate creatine consumption (7–8 mg/kg/day) in the first 1000 days of life may also be critical for the long-term well-being of children, with further analyses from the NHANES study showing a significant relationship between dietary creatine consumption and head circumference in 0–2-year-olds [43].

It is now understood that the pregnant human endometrium, myometrium [44] and placenta [45] can synthesize creatine during pregnancy and are likely significant contributors to providing creatine for the developing fetus and the stability in maternal plasma creatine concentrations observed throughout gestation. Multiple independent studies of placental metabolism in cases of fetal growth restriction and preeclampsia have also identified increased placental creatine concentrations at birth [46,47], and a reliance on the creatine-phosphocreatine system as an oxygen-independent pathway for generating ATP [48]. These data further highlight the importance of creatine for energy generation throughout pregnancy.

Maternal provisions of creatine after birth and the transfer of creatine to the newborn through human milk are also gaining attention. A recent study found creatine concentrations were highest in the colostrum immediately after birth, before decreasing significantly in the first 2-week postpartum. These concentrations were not associated with creatinine or macronutrients, suggesting independent secretion of creatine into milk and a critical demand for creatine immediately after birth that needs to be met by exogenous intake [49]. This raises questions about whether infants solely fed on formula, derived from plant sources devoid of creatine, may become creatine-depleted in the early postnatal period [50], and what the implications may be for newborns, particularly those born prematurely, who often receive total parenteral nutrition devoid of creatine [51].

Dietary creatine supplementation during pregnancy is also being considered as a prophylactic to minimize perinatal injury following episodes of acute hypoxia and infection that often occur *in utero* [52]. Initial studies conducted in the precocial spiny mouse model of birth asphyxia found maternal dietary creatine supplementation at 5% w/w for 18 days from mid-gestation to term resulted in significant improvements in short and long-term offspring survival [53], brain [54], diaphragm [55,56], kidney [57,58], and skeletal muscle health [59]. In the chronic instrumented fetal sheep, long-term creatine supplementation (~13 days) resulted in a five-fold increase in circulating creatine levels and ~ 60% increase in cerebral creatine content in cortical and deep gray matter regions [60]. This effect may be due to creatine’s capacity to mitigate cerebral aerobic and anaerobic metabolism during acute periods of cellular hypoxia [61]. More recently, a study has shown that creatine prophylaxis reduced the incidence of hypoxia-induced electrographic seizures by 60% following an acute mild-to-moderate hypoxic insult [62].

Notably, in fetal sheep studies, increased antenatal exposure to creatine did not cause any significant changes in fetal movement, heart rate, blood pressure, cerebral metabolism (except a reduction in pyruvate and glycerol and gene expression of mitochondrial complex II and IV) or cerebral electroencephalography [60,62–64]. Creatine supplementation from mid-gestation resulted in offspring with increased body weight but also decreased anxiety-like behavior as assessed by the elevated plus maze at postnatal day 28–32 (equivalent to adolescent ages) [65]. Overall, the effects of prolonged antenatal creatine supplementation on *in-utero* cerebral development appear minimal and do not cause any significant disturbances to cognitive behavior in offspring. With a focus on establishing the safety profile of supplementation during pregnancy, creatine continues to present as an efficacious treatment for fetal compromise, with clinical data on dosing and tolerability now being collected to inform potential randomized controlled clinical trials (ACTRN:12620001373965).

### 2.4. Perimenopause: a new era for creatine research

Perimenopause, characterized by fluctuations in female sex hormones, is often accompanied by negative symptoms that can diminish quality of life. Common side effects of perimenopause include metabolic changes that may lead to adverse adaptations in body composition, increases in vasomotor symptoms, and declines in mental health and/or cognition. This transitional period has been identified as a critical period during which creatine supplementation may offer significant benefits. Despite the potential benefits of creatine in this mid-life stage, to date, there is no research directly evaluating the effects of creatine supplementation in perimenopausal women. Some studies to date have included women in their late 50s (Chilibeck et al.) [13], which may have included some women in perimenopause, but hormonal status was not evaluated. The application of creatine in perimenopause is a crucial area to explore based on existing data demonstrating the positive effects of creatine supplementation on lean mass and strength, bone health, reducing fatigue, and mental health in males and in older populations. Areas of opportunity for creatine in perimenopause include:

- **Preserving Muscle Mass and Strength**: During perimenopause, declining estrogen, progesterone, and testosterone levels can lead to a loss of muscle mass and strength [66,67]. Creatine, especially when combined with resistance training, may help mitigate this muscle loss and promote muscle strength during a time when lean mass loss is accelerated at ~ 1.5 lbs per year [68].
- **Improving Bone Health**: Perimenopause can also decrease bone density, increasing the risk of osteoporosis. Creatine may support bone health by enhancing muscle strength and improving balance, which reduces the risk of falls. Research suggests that creatine can positively influence bone mineral density when paired with resistance exercise in older post-menopausal women. Creatine has the potential to support bone health earlier in life (40–50 yrs), and time when bone begins to recede, which could have long-term implications for improving health and quality of life for women.
- **Reducing Fatigue**: Fatigue is one of the most common complaints of women in perimenopause. Creatine can help reduce fatigue by increasing energy availability in cells, which is particularly beneficial during hormonal fluctuations. Supplementation with creatine is also likely to support greater workout intensity and volume, recovery quality, and sleep duration following resistance training, which would indirectly affect muscle, body composition, and bone.
- **Cognitive Function**: Cognitive changes, such as memory lapses and difficulty concentrating, are common during perimenopause. Creatine’s role in ATP regeneration can benefit brain function, potentially improving cognitive performance and reducing mental fatigue.
- **Supporting Mood**: Mood swings and emotional changes are common during perimenopause. Creatine may help support a more stable mood by improving overall energy levels and cognitive function

#### 2.4.1. Mental health & cognitive function

Commonly reported symptoms associated with perimenopause include changes in cognition and mood. These changes can manifest feelings of anxiety and/or depression, “brain fog,” or impaired memory. Previous literature has consistently identified perimenopause as a period of heightened vulnerability to depression [69]. For example, a longitudinal study found that the risk of depression increased during perimenopause, but then declined after the transition to post-menopause [70]. Creatine supplementation has been identified as a method of increasing SSRI efficacy, and thereby decreasing feelings of depression and anxiety [71]. Brain fog is a commonly reported symptom throughout the menopause transition and is characterized by difficulties with memory and/or performing cognitive tasks [72]. Creatine supplementation has been shown to aid in older adults’ memory, with a recent systematic review of 23 randomized-controlled trials finding that creatine improved memory compared to a placebo [73]. This is, namely, thought to be due to the increase in creatine stores in the brain and improving mitochondrial function [73,74]. Therefore, creatine supplementation may be an effective means of decreasing brain fog associated with perimenopause.

### 2.5. Creatine in postmenopause

Creatine supplementation offers significant benefits for postmenopausal women, particularly in maintaining muscle mass, improving strength, supporting bone health, and enhancing cognitive function and mood. Some of the most foundational studies for creatine supplementation in women have been completed in older women (57 yrs+) [13,75–78], assumed to be post-menopausal. These studies indicate that creatine supplementation can significantly improve muscle strength and physical function in postmenopausal women. Short-term, high-dose creatine supplementation (0.3 g/kg/day for 7 days) is effective for increasing muscle mass and strength. These early studies consistently demonstrated that when combined with resistance training, older women can increase strength, lean mass, and muscle function, when combined with creatine supplementation [75,76,79]. More recently, creatine use in post-menopausal women has demonstrated a positive impact on bone health and strength [77], with the longest 2-year randomized controlled study combining creatine supplementation and resistance exercise recently published. Chilibeck et al. demonstrated a positive impact of creatine supplementation on bone geometric properties and lean mass in post-menopausal women [77]. Creatine supplementation in postmenopausal women significantly enhances muscle mass, strength, bone health, and cognitive function, especially when combined with resistance training, as demonstrated by foundational and recent studies.

## 3. Supplementation considerations for creatine

To date, there does not appear to be differences required in the approach to supplementation between men and women, as previously described in the *Dosing Strategies* section (Smith-Ryan et al). More consideration should be given to the goals of supplementation and the time it takes for muscle and brain creatine stores to be elevated. A loading phase, 20 grams per day in 4 × 5-gram doses, is the most efficient way to increase muscle creatine stores, requiring 5–7 days [80]. A similar increase in muscle creatine stores can be realized after a daily 5-gram dose of creatine for 3–4 weeks. This is often the reason why most research studies first employ a loading phase, to more rapidly increase muscle creatine stores in 5–7 days. Following a loading phase, and for a daily dosing strategy, consistency of 3–5 grams per day appears to be an important factor in maintaining tissue saturation. The area of creatine supplementation and brain uptake is still being explored. It does appear that slightly higher doses, 15–20 grams per day for 5–7 days, followed by 5–10 grams is optimal to increase brain creatine concentrations [81].

## 4. Conclusion

Creatine supplementation presents a promising strategy for enhancing various aspects of women’s health across the lifespan. Research on creatine supplementation in women has been ongoing for the last two decades, with several studies initially focused on active and older women. More recently, studies have improved their design to account for the menstrual cycle and female sex hormones. This has enhanced the understanding of creatine supplementation in women and has influenced their application. The evidence suggests that creatine can improve muscle strength, exercise performance, and body composition, particularly when combined with resistance training. Additionally, creatine may offer cognitive and mood benefits, potentially alleviating symptoms of depression and enhancing brain function, which is an important target area for women in different life stages. While the specific effects of creatine may vary depending on hormonal fluctuations and life stages such as pregnancy and menopause, the overall risk-to-benefit ratio of creatine supplements appears to provide more benefit and potential benefit, compared to risks. This may likely hold true for life stages still under investigation. Most notably, during pregnancy. Future research should continue to explore the nuanced impacts of creatine supplementation in women, focusing on optimizing dosing strategies and understanding the long-term implications for health and well-being. A targeted call for action to increase research in the areas of pregnancy and perimenopause is needed.
